# Systematic regulation of circulating lipids, amino acids, and blood glucose to enhance the prevention of hyperthyroidism: A Mendelian randomization analysis

**DOI:** 10.1097/MD.0000000000042912

**Published:** 2025-06-20

**Authors:** Xiao-Hong Wang, Qin Wan

**Affiliations:** a Department of Endocrinology and Metabolism, Wenjiang District People’s Hospital, Chengdu, China; b Department of Endocrinology and Metabolism, Affiliated Hospital of Southwest Medical University, Luzhou, China; c Metabolic Vascular Disease Key Laboratory of Sichuan Province, Luzhou, China; d Sichuan Clinical Research Center for Diabetes and Metabolism, Luzhou, China; e Sichuan Clinical Research Center for Nephropathy, Luzhou, China; f Cardiovascular and Metabolic Diseases Key Laboratory of Luzhou, Luzhou, China.

**Keywords:** amino acids, blood glucose, circulating lipids, hyperthyroidism, Mendelian randomization

## Abstract

Several observational investigations have documented correlations between circulating metabolic biomarkers and hyperthyroidism; nevertheless, the implications of blood lipids, amino acids, and blood glucose in hyperthyroidism remain elusive. Employing summary-level data from the most recent large-scale genome-wide association study(N = 136,016) for 233 circulating metabolic biomarkers, along with data on hyperthyroidism from the R10 dataset released by the FinnGen consortium(N = 412,181), we performed a bidirectional two-sample Mendelian randomization (MR) analysis. We computed the impacts of both utilizing the inverse variance weighted, MR Egger, weighted median, simple mode, and weighted mode techniques, and evaluated the dependability of the findings utilizing Cochran Q test, MR-Egger intercept regression analysis, and MR-PRESSO. Subsequently, a reverse MR analysis was conducted on the circulating metabolic biomarkers identified to exhibit an association with hyperthyroidism in the forward MR analysis. The inverse variance-weighted analysis revealed that for each 1-standard deviation increase in alanine levels, glucose levels, and the cholesteryl esters to total lipids ratio in large very low-density lipoprotein particles, the risk of hyperthyroidism decreased by 14%, 19%, and 15%, respectively. The reverse MR analysis did not identify any significant effect of hyperthyroidism on circulating metabolic biomarkers. Alanine levels, glucose levels, cholesteryl esters to total lipids ratio in large very low-density lipoprotein levels, and the free cholesterol to total lipids ratio in large low-density lipoprotein levels were differentially associated with the risk of hyperthyroidism, and have the potential to be used as biomarkers of hyperthyroidism. The findings of this study may offer novel insights into the prevention and management of hyperthyroidism.

## 1. Introduction

Hyperthyroidism stands as a paramount malady within the endocrine domain, predominantly instigated by Graves’ disease, toxic adenoma, among others,^[1]^ heralded by escalated thyroid hormone levels within tissues, precipitating an array of hallmark symptoms encompassing heat intolerance, palpitations, anxiety, fatigue, muscle weakness, and the like.^[2]^ Typically, the diagnosis of hyperthyroidism manifests relative simplicity, as nearly all instances exhibit elevated serum thyroid hormones coupled with diminished serum thyroid-stimulating hormone levels.^[3]^ Reports indicate that hyperthyroidism afflicts 2.5% of the global adult populace and is intricately linked with heightened susceptibilities to osteoporosis, heart disease, and mortality.^[4]^

Positioned as a pivotal facet within the realm of metabolic disorders, manifold studies have elucidated the intricate interplay between diverse metabolic markers and thyroid function, encompassing entities like sex hormones,^[5]^ smoothelin-like protein 1,^[6]^ and the like Undoubtedly, researchers acknowledge population iodine intake as a preeminent determinant in precipitating thyroid disorders.^[7]^ Empirical evidence suggests that in locales boasting adequate iodine consumption, the prevalence of hyperthyroidism among adults is limited to a mere 0.5%.^[8]^

Current literature underscores an intimate nexus linking hyperthyroidism with metabolic disorders like diabetes, obesity, among others,^[9–11]^ positing the presence of shared underlying pathophysiological mechanisms across these metabolic ailments. Nonetheless, the enigmatic causal linkage between blood glucose, amino acids, and blood lipids – vital circulating metabolites in the context of diabetes and obesity – and hyperthyroidism still eludes elucidation.

Mendelian randomization (MR) stands as a distinguished methodology acknowledged for delineating relationships between exposure and outcome by leveraging genetic variation.^[12]^ Due to the inherent limitations of randomized controlled trials, including substantial workload and high costs, MR is increasingly regarded as an effective alternative.^[13]^ Since genetic variation is arbitrarily distributed at conception, antedating any malady, it holds the potential to mitigate the impact of sundry confounding elements such as the environment.^[14]^ MR has been extensively deployed to investigate the nexus between hyperthyroidism and various other factors, encompassing inflammatory bowel disease,^[15]^ anemia,^[16]^ and coffee consumption,^[17]^ among others. The objective of this investigation is to scrutinize the association between blood glucose, amino acids, and blood lipids and the susceptibility to hyperthyroidism leveraging the most recent published genome-wide association study (GWAS) data comprising 233 circulating metabolic biomarkers.

## 2. Method

### 2.1. Study design

**Figure 1.**
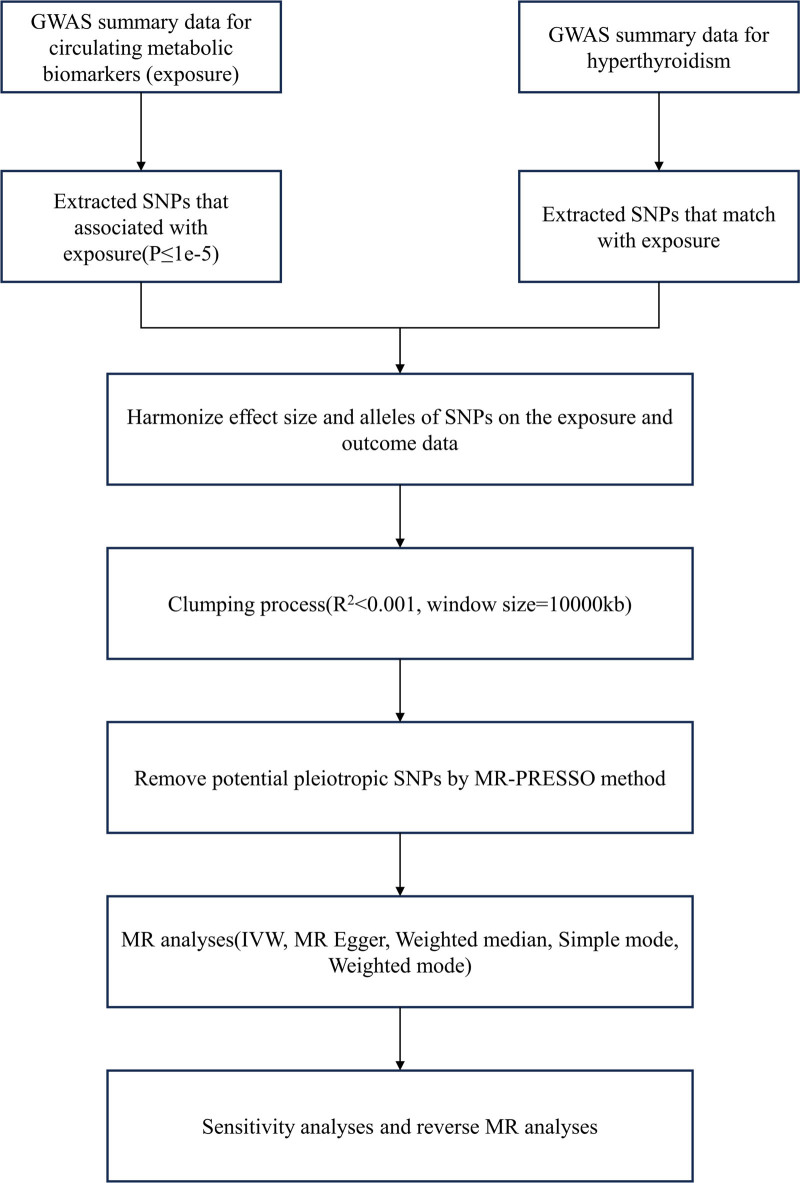
Study design and workflow.

Figure 1 illustrates the schematic representation delineating the study design process. The study employed dual-sample MR to investigate the causal nexus between circulating metabolic biomarkers and the susceptibility to hyperthyroidism. MR investigations must adhere to 3 foundational assumptions.^[18]^ Firstly, genetic variants ought to exhibit close association with the exposure of interest. Secondly, these variants should remain independent of potential confounding variables. Lastly, these genetic instruments should solely influence the outcome through the exposure of interest.

### 2.2. Data sources

The genetic variance of 233 circulating metabolic biomarkers is sourced from the most recent GWAS encompassing 136,016 individuals from 33 cohorts worldwide,^[19]^ predominantly comprising individuals of European descent (N = 120,241). The summary data pertaining to hyperthyroidism GWAS emanates from the R10 dataset published by the FinnGen consortium.^[20]^ The investigation employed pertinent data associated with the “hyperthyroidism” phenotype, encompassing a total of 412,181 participants, comprising 230,310 females and 181,871 males. There is essentially no sample overlap between these 2 different studies on different populations.

### 2.3. Instrumental variable (IV)

This investigation employed the following selection criteria for IVs: (1) single nucleotide polymorphisms (SNPs) linked with diverse circulating metabolic biomarkers, surpassing the genome-wide significance threshold (*P* < 1 × 10^−5^), were identified as prospective IVs^[21]^; (2) for the acquisition of autonomous IVs, SNPs underwent pruning utilizing data derived from the 1000 Genomes Project, thereby eliminating linkage disequilibrium between individual SNPs (*R*^2^ < 0.001 within a 10,000-kb distance); (3) SNPs possessing a minor allele frequency ≤ 0.01 were omitted from consideration; and (4) in the case of palindrome SNPs, the forward strand allele was deduced utilizing allele frequencies.

### 2.4. Statistical analysis

The present investigation employed 5 commonly utilized MR techniques to assess the potential association between 233 circulating metabolic biomarkers and hyperthyroidism. These methods encompassed the inverse variance weighted (IVW), MR Egger, weighted median, simple mode, and weighted mode approaches.^[22,23]^ The IVW method was employed as the primary outcome measure, while the remaining 4 methodologies were utilized as supplementary analyses. The findings derived from the IVW method are typically regarded as the most trustworthy, provided that no horizontal pleiotropy is observed.^[24]^ Cochran Q test was employed to assess the heterogeneity of the results, while leave-one-out analysis was utilized to pinpoint potential heterogeneous SNPs. In the event of heterogeneity in the findings, the outcomes derived from the random-effects model IVW were utilized for analysis. The MR-Egger regression intercept method was employed to evaluate the presence of horizontal pleiotropy. Moreover, MR-PRESSO executed a comprehensive global examination to assess SNPs exhibiting heterogeneity, and subsequently furnished rectified estimates following the removal of outliers.^[25]^ The statistical robustness of IVs was assessed through the computation of the F statistic, employing the subsequent formula:


$$\begin{array}{l} F=\frac{R^{2}\times \left(N-1-K\right)}{\left(1-R^{2}\right)\times K} \end{array}$$

*R*^2^ delineates the proportion of variance in exposure elucidated by genetic variation. N denotes the size of the sample. K denotes the number of instruments.^[26]^ Should the F statistic exceed 10, the presence of significant bias due to weak IVs is deemed negligible.

To evaluate the potential impact of hyperthyroidism on established key circulating metabolic biomarkers. Furthermore, we undertook reverse MR analysis employing SNPs associated with hyperthyroidism as IVs. Sensitivity analyses were conducted employing Cochran Q test, MR-Egger regression intercept method, and MR-PRESSO. All statistical analyses were carried out utilizing the TwosampleMR (version 0.5.6) package within R software (version 4.3.3).

## 3. Result

### 3.1. Genetic instrument selection

SNPs deemed suitable IVs for 233 metabolites were chosen based on IV screening criteria. All selected SNPs exhibit F statistics exceeding 10, suggesting insignificance of bias arising from weak IVs. For detailed information regarding the selected IVs, please consult Table S1, Supplemental Digital Content, https://links.lww.com/MD/P194.

### 3.2. Association between circulating metabolic biomarkers and hyperthyroidism

**Figure 2.**
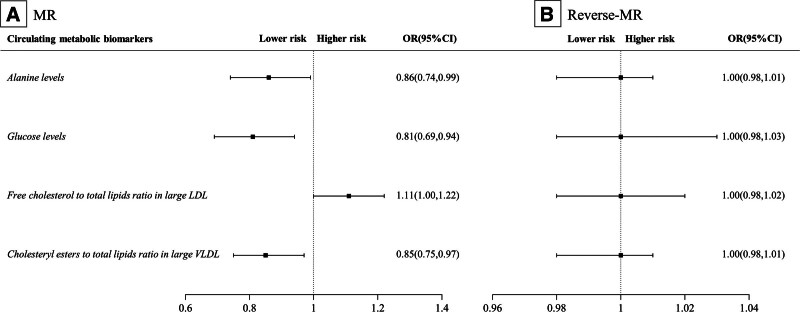
Forest plot of the association between each 1-SD increase in the circulating metabolic biomarkers and the risk of developing hyperthyroidism; alanine levels, glucose levels and cholesteryl esters to total lipids ratio in large VLDL was negatively associated with the risk of hyperthyroidism, whereas free cholesterol to total lipids ratio in large LDL was positively associated with the risk of hyperthyroidism. LDL = low-density lipoprotein, SD = standard deviation, VLDL = very low-density lipoprotein.

Illustrated in Figure 2 and Table S2, Supplemental Digital Content, https://links.lww.com/MD/P194, outcomes derived from the IVW method reveal that elevating circulating levels of alanine, glucose, and cholesteryl esters to total lipids ratio in large very low-density lipoprotein (VLDL) by 1 standard deviation (SD) correlates with a diminished hyperthyroidism risk(*P* < .05), exhibiting ORs of 0.86 (95%CI = 0.74,0.99), 0.81 (95%CI = 0.69,0.94), and 0.85 (95%CI = 0.75,0.97), respectively. Conversely, augmenting Free cholesterol to total lipids ratio in large low-density lipoprotein (LDL) by 1 SD is linked to heightened hyperthyroidism risk, with an OR of 1.11 (95%CI = 1.00, 1.22).

### 3.3. Sensitivity analyses

**Figure 3.**
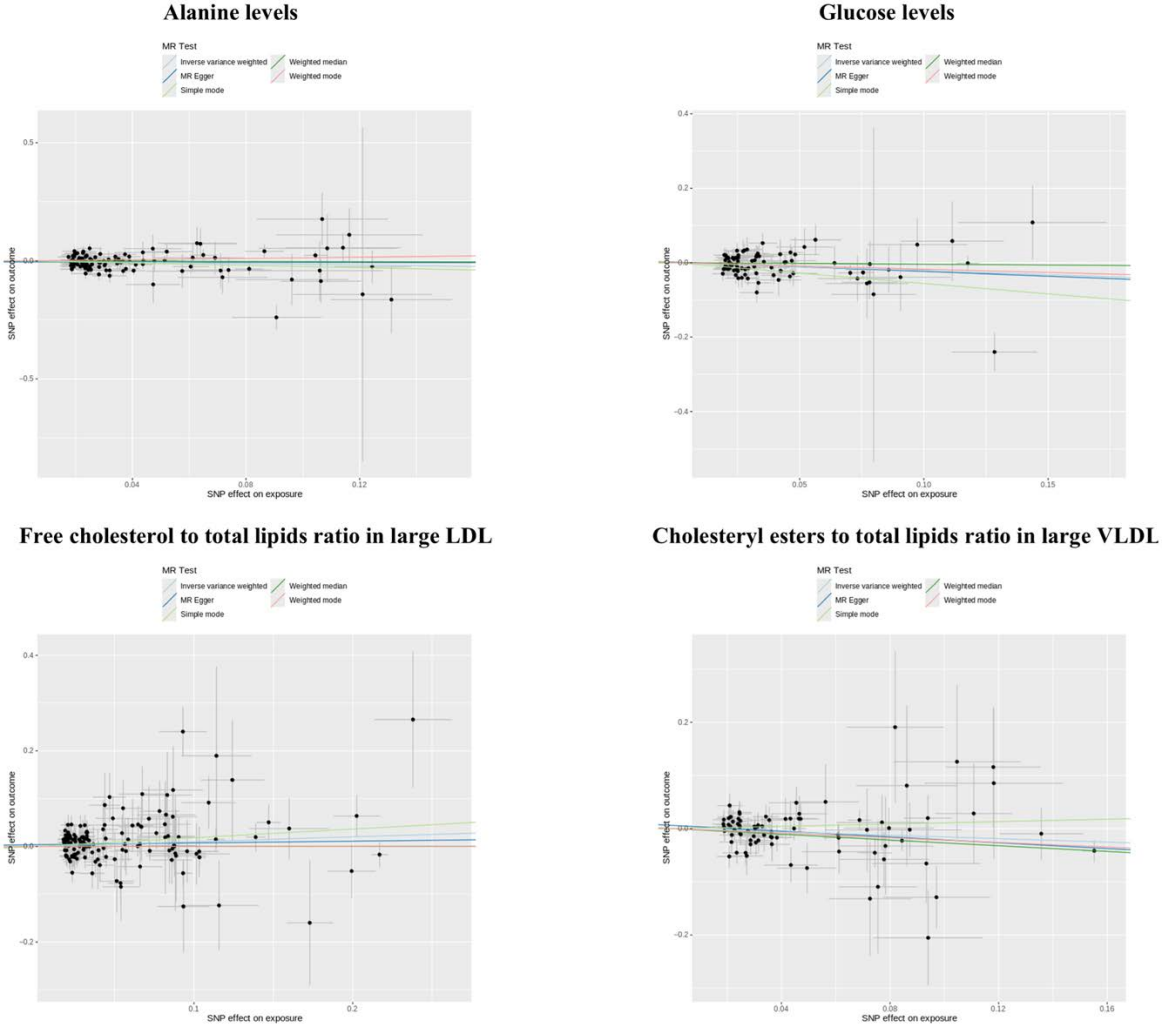
Scatterplots of the causal relationship between circulating metabolic biomarkers and hyperthyroidism by 5 MR statistical methods. IVW analysis was used as the main assessment statistic and the remaining 4 analyses were used as sensitivity analyses. MR = Mendelian randomization, IVW = inverse variance weighted.

**Figure 4.**
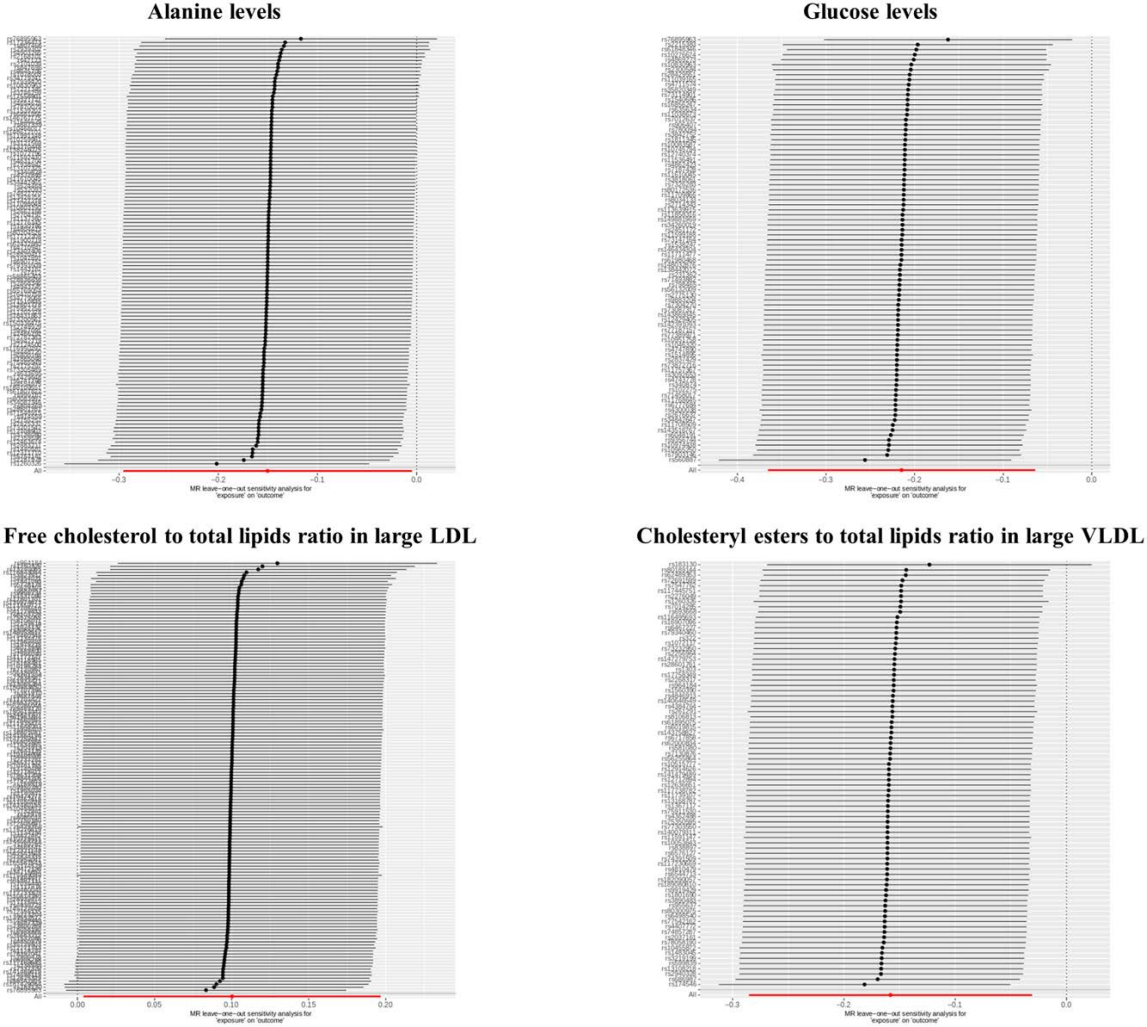
Leave-one-out plots for the causal association between circulating metabolic biomarkers and hyperthyroidism.

Illustrated in Figure 3 and Table S3, Supplemental Digital Content, https://links.lww.com/MD/P194, results obtained from MR Egger, weighted median, simple mode, and weighted mode approaches consistently exhibit positive trends that align well with the IVW test findings. Within the leave-one-out plot (Fig. 4), no conspicuous outliers were detected among the IVs associated with the 4 circulating metabolic biomarkers. As depicted in Table S4, Supplemental Digital Content, https://links.lww.com/MD/P194, Cochran Q test and the MR-PRESSO global test unveil heterogeneity concerning circulating alanine levels (*P* = .02). The remaining 3 circulating metabolic biomarkers exhibit no evident heterogeneity(*P* > .05). Furthermore, upon application of the MR-Egger regression intercept method, no indications of horizontal pleiotropy were discerned across all outcomes(*P* > .05).

### 3.4. Reverse MR

Illustrated in Figure 2 and Tables S4 to S6, Supplemental Digital Content, https://links.lww.com/MD/P194, a total of 35 SNPs were chosen as IVs for hyperthyroidism. All selected SNPs possess F statistics exceeding 10, denoting the insignificance of bias stemming from weak IVs. IVW test outcomes suggest the absence of a reverse association between hyperthyroidism and circulating levels of alanine, glucose, free cholesterol to total lipids ratio in large LDL, and cholesteryl esters to total lipids ratio in large VLDL. Cochran Q test and MR-PRESSO global analysis revealing consistent heterogeneity concerning circulating levels of Alanine, Glucose, and Free cholesterol to total lipids ratio in large LDL. MR-Egger regression intercept analysis yielded no evidence of significant horizontal pleiotropy in the findings (*P* > .05).

## 4. Discussion

In this investigation, we performed a two-sample bidirectional MR analysis employing summary-level GWAS data on 233 circulating metabolic biomarkers recently disseminated by Karjalainen MK et al,^[19]^ alongside summary statistics data pertaining to hyperthyroidism sourced from the FinnGen consortium R10 release. Our findings revealed that elevating circulating alanine levels, glucose levels, and cholesteryl esters to total lipids ratio in large VLDL by 1 SD correlated with a 14% decrease (OR = 0.86), 19% decrease (OR = 0.81), and 15% decrease (OR = 0.85) in hyperthyroidism risk, correspondingly. Conversely, augmenting circulating free cholesterol to total lipids ratio in large LDL by 1 SD was associated with an 11% increase (OR = 1.11) in the risk of hyperthyroidism. Although Cochran Q test suggested potential heterogeneity among some metabolites, the findings from 4 sensitivity analyses, including the weighted median method, were directionally consistent with the IVW results, thereby reinforcing the reliability of the IVW findings.

Alanine, among the 21 amino acids constituting human proteins, exerts specific effects in fostering muscle growth and bolstering immunity.^[27]^ A MR investigation conducted by Huang X et al^[28]^ revealed that elevated plasma alanine levels were linked to a 35% escalation in diabetes risk. Furthermore, a prospective investigation originating from China revealed a positive correlation between plasma concentrations of branched-chain amino acids, aromatic amino acids, aspartate, alanine, glutamate, glycine, 2-aminoadipic acid, histidine, methionine, and proline with the onset of type 2 diabetes mellitus.^[29]^ Findings from these 2 studies suggest that elevated alanine levels might predispose individuals to a heightened risk of diabetes, a metabolic disorder commonly co-occurring with hyperthyroidism. Additionally, an animal investigation observed heightened expression of alanine aminotransferase in the livers of obese and diabetic mice, as well as individuals with type 2 diabetes.^[30]^ Targeted suppression of alanine aminotransferase in hepatic cells demonstrated potential in delaying hyperglycemia onset. A recent 15-year observational study conducted in a middle-aged and elderly Brazilian population reported that impaired thyroid function, as indicated by elevated thyroid hormone levels, was associated with increased alanine concentrations, aligning closely with our findings.^[31]^ On the other hand, alanine plays a role in the synthesis of several antioxidants, such as glutathione, and excessively low levels of alanine may impair antioxidant capacity and influence thyroid function by passing through the follicular cells of the thyroid gland.^[32,33]^ Regrettably, investigations into the correlation between alanine and thyroid function are scarce, necessitating further inquiry into its underlying mechanism via additional foundational experiments.

Over an extensive period, thyroid hormones have been recognized as pivotal regulators of glucose homeostasis.^[34]^ Investigation into the interplay between thyroid hormones and glucose metabolism commenced as far back as a century ago, with the term “thyroid diabetes” coined to delineate the detrimental impact of heightened thyroid hormone levels on glucose homeostasis.^[35]^ Several studies propose an interaction between hyperthyroidism and diabetes mellitus; however, the precise ramifications remain elusive.^[36,37]^

Cholesteryl esters and free cholesterol constitute 2 cholesterol forms within the human body. Upon cholesterol intake, a portion remains unbound, while the remainder undergoes conversion into cholesteryl esters for storage. Current research suggests that heightened free cholesterol levels serve as a significant risk factor for thyroid-associated ophthalmopathy and represent a prevalent extra-thyroidal manifestation of Graves’ disease.^[38–40]^ Additionally, a separate investigation observed that serum free cholesterol levels are notably elevated in individuals with hyperthyroidism compared to non-hyperthyroidism patients.^[41]^ These outcomes align generally with our study findings.

It is important to note that iodine intake varies significantly across global populations, which may influence the capacity of circulating metabolites to regulate thyroid function.^[42]^ An observational study has shown that iodine intake influences glucose and lipid metabolism and is strongly associated with metabolic syndrome.^[43]^ Another study suggested a potential link between low iodine-rich food intake and brain volume atrophy.^[44]^ Fortunately, the genetic variations necessary for MR analysis precede potential confounding factors such as dietary and environmental interventions, which helps minimize bias from these influences.

### 4.1. Strengths and limitations

This investigation possesses several notable strengths. Firstly, through the utilization of MR to explore the interplay between blood glucose, amino acids, blood lipids, and hyperthyroidism, it effectively mitigates biases stemming from confounding factors. Secondly, the genetic markers associated with circulating metabolic biomarkers are sourced from the most recent large-scale GWAS dataset, thereby bolstering the robustness of the chosen IVs. Additionally, various sensitivity analyses and reverse MR analyses were conducted to fortify the dependability of the outcomes. Nonetheless, this study is not without its constraints. The employment of summary-level GWAS data precludes exploration into nonlinear associations and original datasets, potentially constraining the comprehension of the relationships. Moreover, the existing body of research concerning circulating metabolic biomarkers and hyperthyroidism is relatively scant, thereby restricting the depth of discourse surrounding the study findings. While multiple comparisons could potentially introduce bias to the study findings, the application of various statistical methods in this research has ensured the robustness and reliability of the results. Fourth, due to the limitations inherent in GWAS data, applying a significance threshold of 5 × 10^−8^ for SNPs would yield an insufficient number of SNPs for subsequent statistical analyses. Consequently, following recommendations from existing studies, we adopted a significance threshold of *P* < 1 × 10^−5^ for SNPs.^[45]^ Additionally, we utilized the F-statistic and other sensitivity methods to minimize potential biases associated with this decision. Furthermore, this study primarily relies on GWAS data for MR analysis and lacks relevant experimental validation. In future work, we will further investigate the results of this study through basic experiments, including the construction of mouse and cell models for alanine and cholesteryl ester interventions. Finally, since the GWAS data employed in this study are derived from European populations, caution is advised when generalizing the results to other populations.

## 5. Conclusions

In essence, this bidirectional MR study revealed that circulating alanine levels, glucose concentrations, and cholesteryl esters to total lipids ratio in large VLDL exhibit a protective influence on hyperthyroidism, whereas elevated free cholesterol to total lipids ratio in large LDL serves as a predisposing factor for hyperthyroidism. These findings hold potential implications for clinical practitioners, aiding in more effective early prevention and management strategies for hyperthyroidism.

## Acknowledgments

We are grateful to the authors and participants of all GWASs for the summary statistics used.

## Author contributions

**Formal analysis:** Xiao-Hong Wang.

**Funding acquisition:** Qin Wan.

**Investigation:** Xiao-Hong Wang.

**Methodology:** Xiao-Hong Wang.

**Validation:** Qin Wan.

**Writing – original draft:** Xiao-Hong Wang.

**Writing – review & editing:** Qin Wan.

## Supplementary Material


